# Contralateral breast cancer risk is influenced by the age at onset in BRCA1-associated breast cancer

**DOI:** 10.1054/bjoc.2000.1239

**Published:** 2000-07-03

**Authors:** L C Verhoog, C T M Brekelmans, C Seynaeve, E J Meijers-Heijboer, J G M Klijn

**Affiliations:** Family Cancer Clinic, Department of Medical Oncology, Daniel den Hoed Cancer Centre, University Hospital Rotterdam, Groene Hilledijk 301, Rotterdam, EA, 3075; Department of Clinical Genetics, Erasmus University Rotterdam, The Netherlands

**Keywords:** BRCA1

## Abstract

BRCA1/2 mutation carriers diagnosed with breast cancer have a strongly elevated life-time risk of developing a contralateral tumour. We studied the contralateral breast cancer risk in 164 patients from 83 families with a proven BRCA1 mutation in relation to the age at diagnosis of the first primary breast cancer. In the actuarial outcomes after 10 years’ follow-up, 40% of the 124 BRCA1-patients diagnosed with breast cancer < 50 years had developed contralateral breast cancer, vs 12% of the 40 patients > 50 years at first diagnosis (*P*_logrank_= 0.02). These data suggest that age at diagnosis of the first tumour should be taken into account when prophylactic mastectomy in BRCA1-patients is considered. © 2000 Cancer Research Campaign


*British Journal of Cancer* (2000) **83**(3), 384–386
© 2000 Cancer Research Campaign
doi: 10.1054/ bjoc.2000.1239, available online at http://www.idealibrary.com on IDEAL®

### Contralateral breast cancer risk is influenced by the age at onset in BRCA1-associated breast cancer

**LC Verhoog[1], CTM Brekelmans[1], C Seynaeve[1], EJ Meijers-Heijboer[2] and JGM Klijn[1]**

[1]Family Cancer Clinic, Department of Medical Oncology, Daniel den Hoed Cancer Centre, University Hospital Rotterdam, Groene Hilledijk 301, 3075 EA Rotterdam; [2]Department of Clinical Genetics, Erasmus University Rotterdam, The Netherlands

**Summary** BRCA1/2 mutation carriers diagnosed with breast cancer have a strongly elevated life-time risk of developing a contralateral tumour. We studied the contralateral breast cancer risk in 164 patients from 83 families with a proven BRCA1 mutation in relation to the age at diagnosis of the first primary breast cancer. In the actuarial outcomes after 10 years' follow-up, 40% of the 124 BRCA1-patients diagnosed with breast cancer < 50 years had developed contralateral breast cancer, vs 12% of the 40 patients > 50 years at first diagnosis ($P_{logrank}$ = 0.02). These data suggest that age at diagnosis of the first tumour should be taken into account when prophylactic mastectomy in BRCA1-patients is considered. © 2000 Cancer Research Campaign

**Keywords:** BRCA1

It has long been recognized that familial breast cancer is associated with increased bilateral occurrence of this disease (Anderson, 1971). The identification of the BRCA1 and BRCA2 genes has made it possible to more accurately determine hereditary breast cancer risk for women who carry a germline mutation in one of these genes. In BRCA1 mutation carriers there is a strongly elevated life-time risk of breast cancer while the risk of contralateral breast cancer has been estimated to be as high as 64% (Ford et al, 1994). We previously reported that the rate of metachronous contralateral disease in a group of BRCA1-associated breast cancer patients ($n$ = 49) was 19% after 5 years of follow-up, while synchronous presentation occurred in 4% of the patients (Verhoog et al, 1998). More recently, Robson and colleagues found that in a group of 30 BRCA1/2 mutation carriers (diagnosed with breast cancer before the age of 42) 31% developed contralateral breast cancer within 5 years (Robson et al, 1998).

Because of the high risk of a second primary breast cancer, affected BRCA1 mutation carriers and their physicians may have to face the decision about more radical surgery, including contralateral prophylactic mastectomy at the time of, or in the years following, diagnosis of the first tumour. Therefore, it is of interest to identify any subgroup of patients with hereditary breast cancer which is particularly at risk of developing contralateral breast cancer. With respect to the general population, the risk of contralateral disease is clearly correlated with a younger age at onset of the disease (Prior and Waterhouse, 1978; Hislop et al, 1984; Adami et al, 1985; Broët et al, 1995). This, however, could primarily result from the fact that a larger proportion of breast cancer occurring at a younger age is attributable to genetic susceptibility. We investigated the risk of developing contralateral breast cancer in relationship to age at diagnosis of the first breast cancer, in women from families with an identified BRCA1 mutation.

*Received 30 November 1999*
*Revised 10 March 2000*
*Accepted 10 March 2000*

*Correspondence to:* JGM Klijn

### PATIENTS AND METHODS

Families were identified through the Family Cancer Clinic of the Daniel den Hoed Cancer Centre and the Department of Clinical Genetics of the Erasmus University Rotterdam. The 83 families that were eligible for this study consisted of consecutive unselected families with an identified germline mutation in the BRCA1 gene. In total 24 different BRCA1 mutations were identified. The presence of a BRCA1 mutation was detected by protein truncation test, by allele-specific oligonucleotide hybridization for distinct mutations or by PCR-analysis specific for two large genomic deletions (Hogervorst et al, 1995; Petrij-Bosch et al, 1997). Whenever possible, histological and clinical data from breast cancer patients were collected through hospital records and pathology reports.

Follow-up data of 179 women with histologically confirmed breast cancer from these families were available. In 129 of these 179 patients, a BRCA1 mutation was proven by direct DNA-testing in blood cells or tumour material or because of their position in the pedigree. Six breast cancer patients who were identified as not carrying the familial BRCA1 mutation were excluded. Of the remaining 173 breast cancer patients, nine patients were excluded either because they were diagnosed with synchronous bilateral breast cancer ($n$ = 4; 2%) or because they underwent bilateral mastectomy at the time of diagnosis of the first primary ($n$ = 5); finally 164 patients were evaluated.

The date of a second contralateral primary breast tumour, date of bilateral mastectomy and date of death were selected as end-points. Occurrence of a second contralateral breast cancer was studied in relation to the age of onset of the first primary breast cancer (Tables 1 and 2). Incidence rates and 95% confidence intervals were calculated assuming a Poisson distribution. Finally the cut-off age at first diagnosis of 50 years was chosen because this age is frequently used to make the distinction between premenopausal vs postmenopausal breast cancer (Early Breast Cancer Trialists' Collaborative Group, 1998). Kaplan–Meier probabilities were computed with respect to the contralateral breast cancer-free and overall survival and differences were tested by the logrank test.

**Table 1** Actuarial contralateral breast cancer risk for 164 BRCA1-associated breast cancer patients in relation to the age at diagnosis of the first primary breast cancer (BC)

| Age at first BC | n (%) of patients | Follow-up 2-year | 5-year | 10-year |
|---|---|---|---|---|
| ≤40 | 74 (45) | 0.08 | 0.21 | 0.27 |
| 41–50 | 50 (30) | 0.09 | 0.33 | 0.52 |
| 51–60 | 29 (18) | 0.04 | 0.04 | 0.15 |
| > 60 | 11 (7) | 0.00 | 0.00 | 0.00 |

**Table 2** Annual incidence of contralateral breast cancer (CBC) in BRCA1-associated breast cancer patients in relation to the age at diagnosis of the first primary breast cancer (BC)

| Age at first BC | Woman-years at risk | CBC (n) | Incidence per year of CBC (95% CI) |
|---|---|---|---|
| ≤ 40 | 419 | 19 | 4.5% (2.9–7.1) |
| 41–50 | 268 | 17 | 6.3% (3.7–10.6) |
| 51–60 | 184 | 3 | 1.6% (0.4–4.8) |
| >60 | 56 | 0 | 0 (0–6.6) |

### RESULTS

Median age at diagnosis of the 164 patients with unilateral breast cancer and follow-up was 41 years (range 22–80). The four women (2%) with synchronous bilateral breast cancer that were excluded from the analyses presented with breast cancer at the ages of 33, 34, 34 and 51 years. Table 1 shows the number of patients diagnosed with breast cancer < 40 years, at 41–50 years, 51–60 years, and > 60 years, with the 2-, 5- and 10-year contralateral breast cancer probabilities for the different age-groups. The annual incidence of contralateral breast cancer in relation to age of diagnosis of the first primary is listed in Table 2 as well as the number contralateral cancers that occurred and the number of women years at risk. No contralateral cancers were observed in the 11 patients that were older than 60 years at time of diagnosis of the first breast cancer.

Forty (24%) of the 164 patients were diagnosed with their first breast cancer after the age of 50. Thirty-six (29%) of the remaining 124 patients diagnosed before the age of 50 years developed metachronous contralateral breast cancer vs three (7.5%) of the 40 patients over the age of 50 on diagnosis ($P$ = 0.005). Median follow-up of all 164 patients was 47 months and the overall survivall at 5 and 10 years was 66% and 50%, respectively. Between the two age groups there was no significant difference in overall survival ($P_{\text{logrank}}$ = 0.98, Figure 1).

In the actuarial outcomes after 5 and 10 years follow-up, metachronous contralateral breast cancer was found in 24% and 34%, respectively, of all BRCA1-associated breast cancer patients. In the group of patients diagnosed with breast cancer before the age of 50, contralateral breast cancer after 5 and 10 years follow-up was seen in 30% and 40%, respectively, vs 4% and 12% respectively in BRCA1-associated breast cancer patients older than 50 years at first diagnosis ($P_{\text{logrank}}$ = 0.02, Figure 2).

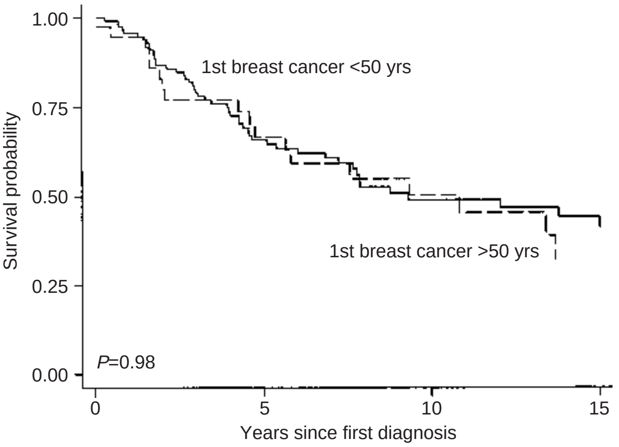

**Figure 1** Overall survivall for BRCA1-associated breast cancer patients (< 50 years at first diagnosis, $n$ = 124, continuous line; > 50 years at first diagnosis, $n$ = 40, dashed line).

### DISCUSSION

In the general population, young age at onset of first breast cancer has been shown to be a risk factor for the occurrence of contralateral breast cancer. However the proportion of breast cancer due to genetic susceptibility will also correlate with a younger age at onset of the disease. With regard to the contribution of BRCA1 mutations to breast cancer in the general population it has previously been estimated that 5.3% of the women diagnosed before the age of 40 carry this mutation, compared to only 1.1% of those aged 50–70 at diagnosis (Ford et al, 1995). Population-based studies in early-onset breast cancer patients confirm these results (Newman et al 1998; Peto et al, 1999). Therefore, young age at onset as a risk-factor for contralateral breast cancer could at least partly be due to the fact that early-onset breast cancer is more frequently the result of genetic susceptibility, whereas a woman with breast cancer resulting from genetic susceptibility could be at an increased risk of developing contralateral breast cancer regardless of the age at onset of her disease. In the present study we show that with respect to BRCA1-associated breast cancer this risk is also correlated with the age at onset of the first breast cancer.

We are not sure of the relevance of these results to the management of contralateral breast cancer risk in BRCA2-associated breast cancer patients, or patients with a strong family history for the disease in general. Earlier we reported that the 5-year rate of contralateral breast cancer in BRCA2 mutation carriers was 12% (Verhoog et al, 1999). In the present study the rate in BRCA1 mutation-carriers is approximately twice that. Our present results are well in line with studies showing that the breast cancer risk in BRCA1 carriers declines with age (Easton et al, 1995). Although the penetrance at ages < 50 years is less for BRCA2 mutations, the life-time penetrance for breast cancer due to BRCA2 mutations may be equal to that of BRCA1 mutation carriers (Ford et al, 1998). This could indicate that, in BRCA2 mutation carriers, the risk for developing contralateral breast cancer does not diminish after the menopause.

Because it was not possible to test all the breast cancer patients from families with an identified BRCA1 mutation for the presence of such a specific mutation, it cannot be ruled out that a few of the patients in our series are in fact sporadic cases. However, restriction to proven BRCA1 carriers did not essentially alter the

© 2000 Cancer Research Campaign

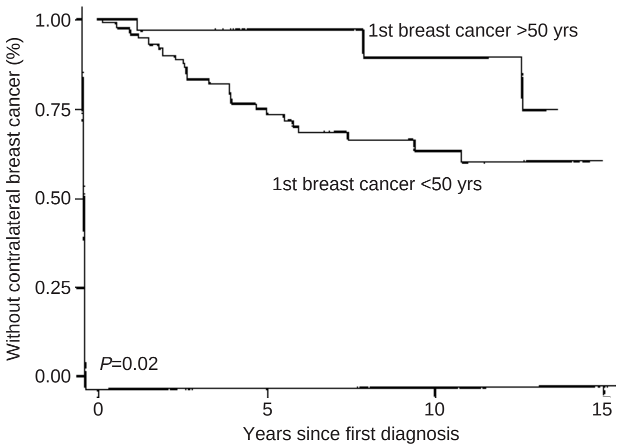

**Figure 2** Proportion of BRCA1-associated breast cancer patients without contralateral breast cancer.

observed trend although numbers were to small to reach statistical significance.

Since the 1980s breast cancer in postmenopausal women with axillary lymph node involvement is generally treated with adjuvant hormonal therapy, i.e. tamoxifen for 2–5 years while 4–6 courses of adjuvant chemotherapy are preferentially used in premenopausal patients. Long-term adjuvant treatment with tamoxifen resulted in a 45% reduction of the risk of contralateral breast cancer (Early Breast Cancer Trialists' Collaborative Group, 1998). This might partly explain the lower rate of contralateral breast cancer in women diagnosed after the age of, 50. In our series of 40 postmenopausal patients, information on the use of antiestrogens was available for 36 patients; only six received adjuvant tamoxifen therapy. Exclusion of these six women did not essentially alter the observed trend, which makes this therapy an unlikely explanation of the observed difference in the rate of contralateral breast cancer between the two age-groups in our study.

In conclusion, these results suggest that apart from the stage of the primary tumour, and time that has elapsed since the onset of disease, age at diagnosis of the first breast cancer in BRCA1 gene-mutation carriers should be taken into account in the decision-making process with respect to contralateral prophylactic mastectomy.

### ACKNOWLEDGEMENT

This study was supported by the Dutch Cancer Society (grant 95–953).

### REFERENCES

Adami HO, Bergstrom R and Hansen J (1985) Age at first primary as a determinant of the incidence of bilateral breast cancer. Cumulative and relative risks in a population-based case-control study. *Cancer* **55**: 643–647

Anderson DE (1971) Some characteristics of familial breast cancer. *Cancer* **28**: 1500–1505

Broët P, de la Rochefordière, Scholl SM, Fourquet A, Mosseri V, Durand J-C, Pouillart P and Asselain B (1995) Contralateral breast cancer: annual incidence and risk parameters. *J Clin Oncol* **13**: 1578–1583

Early Breast Cancer Trialists' Collaborative Group (1998) Tamoxifen for early breast cancer: an overview of the randomised trials. *Lancet* **351**: 1451–1467

Easton DF, Ford D, Bishop DT, and the Breast Cancer Linkage Consortium (1995) Breast and ovarian cancer incidence in BRCA1-mutation carriers. *Am J Hum Genet* **56**: 265–271

Ford D, Easton DF, Bishop DT, Narod SA, Goldgar DE and the Breast Cancer Linkage Consortium (1994) Risk of cancer in BRCA1 mutation carriers. *Lancet* **343**: 692–695

Ford D, Easton DF and Peto J (1995) Estimates of the gene frequency of BRCA1 and its contribution to breast and ovarian cancer incidence. *Am J Hum Genet* **57**: 1457–1462

Ford D, Easton DF, Stratton M, Narod S, Goldgar D, Devilee P, Bishop T, Weber B, Lenoir G, Chang-Claude J, Sobol H, Teare MD, Struewing J, Arason A, Scherneck S, Peto J, Rebbeck TR, Tonin P, Neuhausen S, Barkardottir R, Eyfjord J, Lynch H, Ponder BAJ, Gayther SA, Birch JM, Lindblom A, Stoppa-Lyonnet D, Bignon Y, Borg A, Hamann U, Haites N, Scott RJ, Maugard CM, Vasen H, Seitz S, Cannon-Albright LA, Schofield A, Zelada-Hedman M and the Breast Cancer Linkage Consortium (1998) Genetic heterogeneity and penetrance analysis of the BRCA1 and BRCA2 genes in breast cancer families. *Am J Hum Genet* **62**: 676–689

Hislop TG, Elwood JM, Coldmann AJ, Spinelli JJ, Worth JJ and Ellison LG (1984) Second primary cancer of the breast: incidence and risk factors. *Br J Cancer* **49**: 70–85

Hogervorst FBL, Cornelis RS, Bout M, van Vliet M, Oosterwijk JC, Olmer R, Bakker B, Klijn JGM, Vasen HFA, Meijers-Heijboer H, Menko FH, Cornelisse CJ, den Dunnen JT, Devilee P and van Ommen GJB (1995) Rapid detection of BRCA1 mutations by the protein truncation test. *Nat Genet* **10**: 208–212

Newman B, Mu H, Butler LM, Millikan RC, Moorman PG and King MC (1998) Frequency of breast cancer attributable to BRCA in a population-based series of American women. *JAMA* **279**: 915–921

Peto J, Collins N, Barfoot R, Seal S, Warren W, Rahman N, Easton DF, Evans C, Deacon J and Stratton MR (1999) Prevalence of BRCA1 and BRCA2 gene mutations in patients with early-onset breast cancer. *J Natl Cancer Inst* **91**: 943–949

Petrij-Bosch A, Peelen T, van Vliet M, van Eijk R, Olmer R, Drüsedau M, Hogervorst FLB, Hageman S, Arts PJW, Ligtenberg MJL, Meijers-Heijboer H, Klijn JGM, Vasen HFA, Cornelisse CJ, van't Veer LJ, Bakker E, van Ommen GJB and Devilee P (1997) BRCA1 genomic deletions are major founder mutations in Dutch breast cancer patients. *Nat Genet* **17**: 341–345

Prior P and Waterhouse JA (1978) Incidence of bilateral tumours in a population-based series of breast-cancer patients. I. Two approaches to an epidemiological analysis. *Br J Cancer* **37**: 620–634

Robson M, Gilewski T, Haas B, Levin D, Borgen P, Rajan P, Hirschaut Y, Pressman P, Rosen PP, Lesser ML, Norton L and Offit K (1998) BRCA-associated breast cancer in young women. *J Clin Oncol* **16**: 1642–1649

Verhoog LC, Brekelmans CTM, Seynaeve C, van den Bosch LMC, Dahmen G, van Geel AN, Tilanus-Linthorst MMA, Bartels CCM, Wagner A, van den Ouweland A, Devilee P, Meijers-Heijboer EJ and Klijn JGM (1998) Survival and tumour characteristics of breast-cancer patients with germline mutations in BRCA1. *Lancet* **351**: 316–321

Verhoog LC, Brekelmans CTM, Seynaeve C, Dahmen G, van Geel AN, Bartels CCM, Tilanus-Linthorst MMA, Wagner A, Devilee P, Halley DJJ, van den Ouweland AMW, Meijers-Heijboer EJ and Klijn JGM (1999) Survival in hereditary breast cancer associated with germline mutations of BRCA2. *J Clin Oncol* **17**: 3396–3402

 © 2000 Cancer Research Campaign
